# Resultados Perinatais e Seguimento em Longo Prazo de Tumores Cardíacos Fetais: Estudo de Coorte Histórica de 30 Anos

**DOI:** 10.36660/abc.20220469

**Published:** 2024-03-13

**Authors:** Fabricio Marcondes Camargo, Maria de Lourdes Brizot, Rossana Pulcineli Vieira Francisco, Werther Brunow de Carvalho, Nana Miura Ikari, Stella Verzinhasse Peres, Marco Antônio Borges Lopes, Lilian Maria Lopes

**Affiliations:** 1 Universidade de São Paulo Faculdade de Medicina Hospital das Clínicas São Paulo SP Brasil Hospital das Clínicas da Faculdade de Medicina da Universidade de São Paulo, São Paulo, SP – Brasil; 2 Universidade de São Paulo Faculdade de Medicina Hospital das Clínicas São Paulo SP Brasil Instituto do Coração do Hospital das Clínicas da Faculdade de Medicina da Universidade de São Paulo, São Paulo, SP – Brasil; 3 Universidade de São Paulo Faculdade de medicina São Paulo SP Brasil Universidade de São Paulo – Faculdade de medicina, São Paulo, SP – Brasil; 4 Cardiologia e Ecocardiografia Fetal Pediátrica e Materna São Paulo SP Brasil ECOKID – Cardiologia e Ecocardiografia Fetal Pediátrica e Materna, São Paulo, SP – Brasil

**Keywords:** Neoplasias Cardíacas, Ecocardiografia, Diagnóstico Pré-Natal, Rabdomioma

## Abstract

**Fundamento:**

Seguimento de coorte retrospectiva de 30 anos que se aproxima da história natural dos tumores cardíacos diagnosticados no feto uma vez que nenhum caso foi submetido à interrupção da gestação.

**Objetivo:**

Avaliar a morbidade e mortalidade perinatal e em longo prazo em fetos com diagnóstico de tumor cardíaco. Como objetivo secundário avaliar os fatores que influenciaram os resultados perinatais e pós-natais.

**Método:**

Estudo de coorte retrospectiva envolvendo 74 gestantes com diagnóstico ecocardiográfico fetal de tumor cardíaco acompanhadas em dois serviços de referência no período de maio de 1991 a novembro de 2021. Foi realizada análise descritiva dos dados por meio de frequências absolutas (n) e relativas (%), mediana e intervalos interquartis. Para avaliar a associação entre as características ecocardiográficas e as manifestações clínicas com os resultados perinatais e pós-natais, foi aplicado o teste exato de Fisher. O cálculo da sobrevida global foi realizado pelo método de Kaplan-Meier e a comparação de curvas pelo teste de *log-rank* . O tempo de seguimento, calculado em meses, foi definido a partir da data de alta do hospital à data do status atual (vivo/censura ou óbito). O nível de significância considerado foi de 5% (p<0,05).

**Resultados:**

o rabdomioma é o tipo mais frequente (85%) de tumor cardíaco; apresenta alta morbidade (79,3%) e mortalidade geral de 17,4%; a presença de hidropisia fetal preditiva de óbito.

**Conclusão:**

A presença de hidropisia fetal teve impacto na mortalidade, sendo fator importante para aconselhamento e estabelecimento de prognóstico. A maioria dos óbitos ocorrem antes da alta hospitalar.

**Figure:**
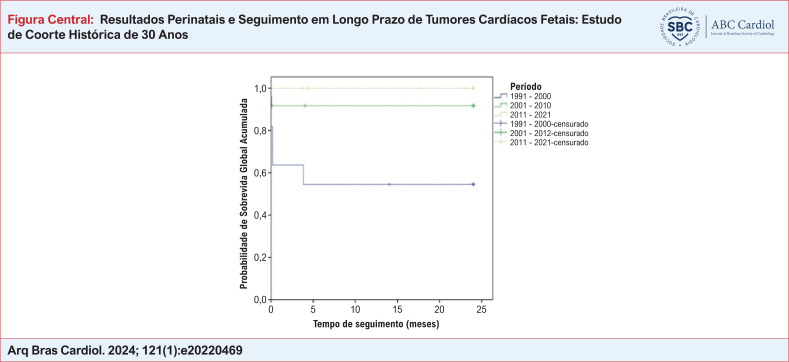


## Introdução

Tumores cardíacos primários são raramente observados no útero ou no pós-natal. A incidência é de 0,009% em rastreamentos ultrassonográficos de baixo e alto risco, e aumenta para 0,2% em centros de referência em cardiologia pediátrica.^1^

Mais de 90% dos tumores cardíacos primários são benignos e podem se originar do pericárdio ou do miocárdio.^2^ Dos cinco tipos histológicos de tumores cardíacos congênitos, os rabdomiomas são responsáveis por 60 a 86% dos tumores, seguidos por teratomas e fibromas, enquanto hemangiomas e hamartomas são extremamente raros.^3^

A presença de tumores cardíacos no feto pode levar a acometimentos hemodinâmicos significativos que influenciam na morbidade e na mortalidade fetal e pós-natal, como hidropisia, arritmia e obstrução aos fluxos de via de entrada e de saída ventricular.^4 - 7^ Além disso, a prevalência de esclerose tuberosa associada ao rabdomioma cardíaco fetal, é alta, estimada entre 50 e 80%.^4 , 8 - 13^ Portanto, o achado de tumor cardíaco é importante indicador na investigação de esclerose tuberosa, doença genética que além do acometimento cardíaco apresenta manifestações clínicas neurológicas, renais, oftalmológicas e dermatológicas.^14 , 15^ A morbidade mais significativa, principalmente quando associada à esclerose tuberosa, relaciona-se com as alterações no sistema nervoso central, incluindo crises convulsivas, atraso no desenvolvimento e transtornos do espectro autista.^14^

Na última década, houve um aumento notável no diagnóstico fetal de tumores cardíacos primários. Parte desse aumento pode ser atribuído aos avanços nas técnicas de imagem que conseguem diagnosticar e classificar com alta acurácia os vários tipos histológicos de tumor.^10 , 13 , 15 , 16^

Poucos são os estudos que avaliam os resultados perinatais e em longo prazo dos fetos com tumores cardíacos.^4 , 8 - 12^ O presente estudo tem como objetivo contribuir a preencher esta lacuna, relatando dados referentes à morbidade e mortalidade, bem como os fatores que influenciaram nesses desfechos. Além disso, a grande contribuição desta série é se aproximar da história natural da doença uma vez que nenhum caso de interrupção da gestação foi encontrado.

## Métodos

Estudo de coorte retrospectivo entre maio de 1991 e novembro de 2021, envolvendo dados de fetos com diagnóstico ecocardiográfico de tumor cardíaco examinados em dois centros de referência em Ecocardiografia Fetal (Unidade de Ecocardiografia e Cardiologia Fetal do Departamento de Obstetrícia do Hospital das Clínicas da Faculdade de Medicina da Universidade de São Paulo, HCFMUSP, e Clínica e Instituto de Ensino e pesquisa da Ecokid). Este estudo foi aprovado pelo Comitê de Ética Hospitalar (CAAE: 93022218.9.0000.0068).

Durante o período de 30 anos de estudo, foram avaliadas 30 915 gestantes no Setor de Ecocardiografia Fetal do HCFMUSP e 46 671 gestantes na Clínica Ecokid, com 74 fetos acometidos por tumores cardíacos.

Os critérios de inclusão compreenderam casos fetos que tiveram confirmação pós-natal ecocardiográfica (no caso dos nascidos vivos) ou anatomopatológica (no caso dos óbitos fetais). Nos dois serviços os exames ecocardiográficos foram realizados com o mesmo protocolo de seguimento, uma vez que os profissionais responsáveis pelas avaliações pertenciam aos dois locais. Todos os exames ecocardiográficos foram realizados e revisados por apenas dois profissionais com formação em Ecocardiografia Fetal e formação médica em cardiologia pediátrica (F.M.C e L.M.L.).

**Figura 1:**
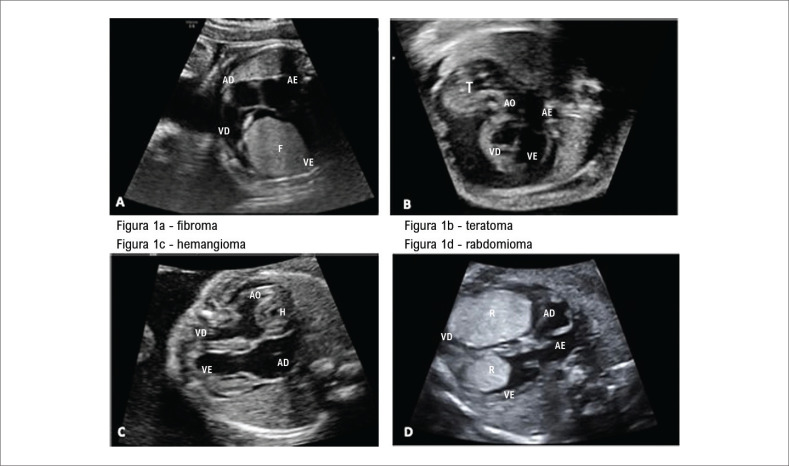
Exemplos dos tipos de tumores cardíacos fetais diagnosticados por ecocardiografia fetal. VD: ventrículo direito; VE: ventrículo esquerdo; AD: átrio direito; AE: átrio esquerdo; AO: aorta.

Os tumores foram classificados de acordo com o aspecto ecocardiográfico em termos de ecogenicidade (homogêneo ou cístico), limites de bordas (delimitadas ou irregulares), assim como número (solitário ou múltiplo), localização e dimensão (maior ou menor que 20mm). Foi considerado o diagnóstico de rabdomioma quando existiam massas de aspecto homogêneo e dimensões variáveis, múltiplas em sua maioria, mas podendo estar solitárias, sem fluxos em seu interior. Por outro lado, foram consideradas como fibroma as massas solitárias hiperecogênicas com calcificação e degeneração cística em seu interior. Os teratomas mostraram ecogenicidade mais complexa, com cistos e partes sólidas mescladas com calcificações e o único caso de hemangioma mostrou, como descrito na literatura, ser vascularizado e localizado em átrio direito. A Figura 1 mostra os exemplos dos tipos de tumores cardíacos fetais diagnosticados por ecocardiografia fetal.

Também foi avaliada a repercussão hemodinâmica do tumor principalmente quanto à presença de hidropisia, obstrução aos fluxos de entrada e de saída do coração e arritmia. A hidropisia fetal foi definida pela presença de derrames em duas ou mais cavidades na ausência de aloimunização.^17^ Para quantificar a insuficiência cardíaca fetal evolutivamente, foi utilizado o escore cardiovascular de James Huhta,^18^ onde foram avaliadas a presença de hidropisia, o índice cardiotorácico, a função cardíaca, o padrão de Doppler arterial e venoso.

Os aparelhos de ultrassom utilizados para os exames ao longo do tempo foram Toshiba 270, HDI 3000 (Advanced Technology Laboratories, Bothell, WA, USA) e aparelhos da marca GE (Voluson 730 Expert, GE Voluson E8, S6 expert e E10). Foram utilizados transdutores setoriais e convexos de 5,0MHz e 3,5MHz. O coração fetal foi examinado por meio de métodos padronizados de imagem bidimensional, Modo M, Doppler espectral (onda pulsada) e fluxo Doppler colorido. O ritmo cardíaco foi determinado por meio da análise da sístole mecânica da parede atrial e ventricular pelo Doppler pulsado.

Após o diagnóstico de tumor fetal, as gestantes cujos fetos não apresentavam acometimento hemodinâmico foram acompanhadas mensalmente. Naquelas em que havia algum acometimento hemodinâmico, como hidropisia e/ou arritmia, o acompanhamento foi semanal ou quinzenal, conforme a gravidade de cada caso. Além disso, as gestantes também foram acompanhadas no pré-natal de alto risco na instituição. O parto era programado para ser realizado com 39 semanas e o tipo de parto seguia a indicação obstétrica.

A confirmação diagnóstica dos tipos de tumores foi realizada no período neonatal por meio de avaliação ecocardiográfica e ressonância nuclear magnética além de análise anatomopatológica nos casos que foram submetidos à cirurgia ou que evoluíram para óbito. Foi utilizada a nomenclatura preconizada no último consenso de classificação de tumores do pericárdio e do coração da Organização Mundial da Saúde, em 2015.^18 , 19^ Os pacientes com diagnóstico confirmado no pós-natal tiveram seguimento ambulatorial com cardiologista pediátrico e os que tiveram diagnóstico de esclerose tuberosa também foram acompanhados por neurologistas pediátricos.

Morbidade foi definida como necessidade de tratamento do paciente em unidade de terapia intensiva, tratamento medicamentoso pós-natal com antiarrítmico, anticonvulsivante ou inibidores da mTOR (everolimus ou sirolimus), necessidade de cirurgia pós-natal, acometimento neurológico, e diagnóstico de esclerose tuberosa. Foi considerado acometimento neurológico a presença de uma das seguintes condições: esclerose tuberosa, crises convulsivas e atraso no neurodesenvolvimento. Os diagnósticos de esclerose tuberosa, crise convulsiva e atraso no neurodesenvolvimento foram obtidos do prontuário e na entrevista, pela ficha de acompanhamento.

### Análise estatística

Foi realizada análise descritiva dos dados por meio de frequências absolutas (n) e relativas (%). O denominador indica o número de casos em que foram obtidas informações àquela variável.

Para análise inferencial dos dados foram incluídos apenas os fetos com rabdomioma. A distribuição das variáveis quantitativas foi analisada pelo teste de Komolgorov-Smirnov para verificar a normalidade e, como os dados não apresentaram distribuição normal, os dados foram descritos em valores medianos e intervalos interquartis. O teste pela Binomial foi aplicado para identificar a diferença entre as proporções dos sexos. Na comparação entre as variáveis independentes em relação aos resultados de óbito e esclerose tuberosa, foi aplicado o teste exato de Fisher. O cálculo da sobrevida global (SG) foi realizado pelo método de Kaplan-Meier e a comparação de curvas pelo teste de *log-rank* . O tempo de seguimento calculado em meses foi definido a partir da data de alta do hospital à data do status atual (vivo/censura ou óbito).

O nível de significância considerado foi de 5% (p<0,05). Os dados foram analisados no programa SPSS versão 20 para Windows.

## Resultados

As indicações das avaliações ecocardiográficas na ocasião do diagnóstico de rabdomioma fetal foram: suspeita de tumor (n=42, 68,8%), arritmia fetal (n=9, 14,7%), suspeita de cardiopatia congênita (n=7, 11,5%), exame de rotina (n=3,5%).

Dos 74 casos descritos, houve confirmação histológica em 12 casos (16,2%), 7 deles após o óbito e dois deles após cirurgia. Entretanto, a ausência de estudo histológico não invalida a confirmação diagnóstica, uma vez que os estudos demonstram que o diagnóstico é realizado em sua grande maioria pelos exames de imagem, clínico e genética.^20^ Além disso, a realização de um procedimento invasivo para diagnóstico não é isento de riscos.

Foram encontrados 11 casos agrupados como formas raras: seis (8,1%) fibromas, quatro (6,4%) teratomas e um (1,3%) hemangioma. Os rabdomiomas foram o mais frequente, somando um total de 63 fetos (85,1%).

### Fibroma

Seis casos de fibroma foram confirmados, dois destes evoluíram para óbito. Em ambos os casos, o tumor era grande, localizado na parede posterior do ventrículo esquerdo. O primeiro faleceu com um ano de vida aguardando transplante cardíaco e o segundo evoluiu para óbito com três dias de vida ( Figura 1.a ), antes da alta hospitalar. Os outros quatro casos estão vivos, não realizaram cirurgia e continuam em seguimento clínico.

### Teratoma

Dos quatro casos de teratoma, três evoluíram para óbito, dois fetais e um neonatal. Todos apresentavam derrame pericárdico importante e hidropisia fetal, sendo um deles submetido à punção do derrame, que provocou expansão da massa tumoral e óbito dois dias após o procedimento, com 25 semanas de gestação. Um paciente encontra-se vivo e assintomático após exérese do tumor ( Figura 1.b ).

### Hemangioma

**Figura 2:**
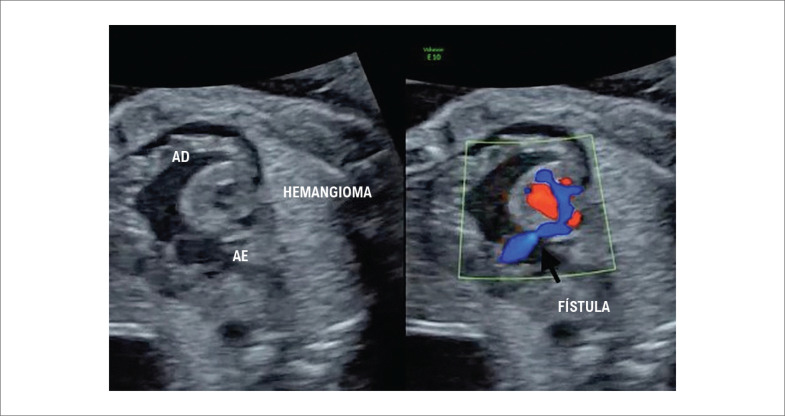
Hemangioma no átrio direito nutrido por uma fistula proveniente da artéria coronária esquerda. AD: átrio direito; AE: átrio esquerdo.

O único caso de hemangioma foi diagnosticado com 24 semanas de gestação, nasceu com 38 semanas em hospital referência em cirurgia cardíaca infantil. Era um tumor vascular localizado no átrio direito ( Figura 1.c ), nutrido por uma fístula proveniente da artéria coronária esquerda ( Figura 2 ). O paciente está bem, em seguimento contínuo com cardiologista pediátrico, sem medicações.

### Rabdomioma

Os rabdomiomas foram o tipo diagnosticado mais frequente, somando um total de 63 fetos (85,1%). A mediana da idade gestacional na ocasião do diagnóstico do rabdomioma foi de 32 (13 – 39) semanas. A mediana da idade materna na época do diagnóstico foi de 29 (15 – 39) anos. História familiar de esclerose tuberosa esteve presente em 18% (11/63) dos casos com diagnóstico de rabdomioma.

A maioria dos tumores (92,3%) esteve localizado nos ventrículos, eram múltiplos (77%) e, aproximadamente um 45% apresentavam um diâmetro maior que 20mm ( Figura 1.d ). Cinquenta e oito (92%) foram nascidos vivos com mediana da idade gestacional no parto de 38 (37-39) semanas e o tipo de parto mais frequente foi a cesárea, em 81,8% (45/55) dos casos. As frequências de recém-nascidos do sexo masculino e do feminino foram similares, 52,5% (31/59) e 47,5% (28/59) respectivamente (p = 0,79). O escore de Apgar menor ou igual a sete foi observado em 7,7% (4/52) dos nascidos vivos.

Cinquenta pacientes (83,3%) evoluíram com algum tipo de morbidade. A mediana do tempo de internação foi de 10 (5-19) dias (5-19), a maioria (95,7) foi admitido em UTI neonatal. Desse total, 16,1% necessitaram de cirurgia e 88,7% receberam alta.

**Figura 3:**
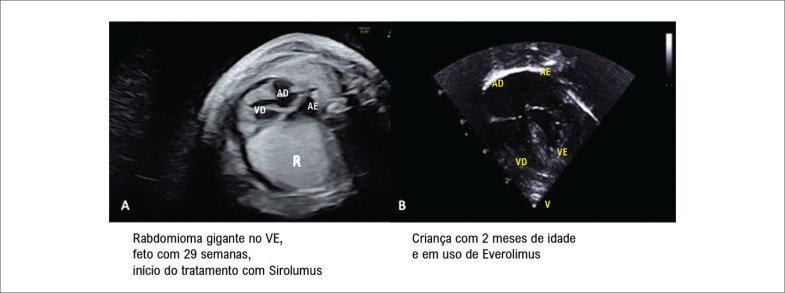
Paciente submetido ao tratamento com inibidor de mTOR antes e depois do nascimento. VD: ventrículo direito; VE: ventrículo esquerdo; AD: átrio direito; AE: átrio esquerdo.

Em relação ao uso de medicação, 51,9% (27/52) dos pacientes necessitaram de tratamento após o nascimento, quatro deles necessitaram de drogas antiarrítmicas, 20 necessitaram de drogas com ação no sistema nervoso central, como anticonvulsivantes e antipsicóticos e três deles receberam inibidores da mTOR, como sirolimus e everolimus ( Figura 3 ). O acometimento neurológico estava registrado no prontuário de 77,8% (35/45) dos pacientes, seja pela presença, muitas vezes concomitante, do atraso do neurodesenvolvimento e de crises convulsivas. Informações sobre o tempo de seguimento foram obtidas em 53 casos com mediana de tempo de seguimento de 70 (12-174,3) meses. A Tabela 1 apresenta a distribuição das características clínicas e ultrassonográficas dos pacientes com diagnóstico ecocardiográfico fetal de rabdomioma.

**Tabela 1:** Distribuição das características clínicas e ultrassonográficas dos pacientes com diagnóstico ecocardiográfico fetal de rabdomioma

Características		N, %
**Sexo**		
	Feminino	28/59 (47,5)
	Masculino	31/59 (52,5)
**Histórico familiar**		11/63 (17,5)
	Hidropisia	9/63 (14,3)
	Arritmia	13/49 (26,5)
**Número de tumores**		
	Único	14/61 (23,0)
	Múltiplos	47/61 (77,0)
**Esclerose tuberosa**		32/44 (72,7)
**Localização**		
	Atrial	4/63 (6,3)
	Ventricular	58/63 (92,1)
	Atrial e ventricular	1/63 (1,6)
**Diâmetro > 20mm**		24/54 (44,4)
**Desfecho**		
	Vivo	52/63 (82,5)
	Óbito *	11/63 (17,5)

O denominador indica o número de casos em que foram obtidas informações referentes à variável.

* Incluídos óbitos fetais, óbitos neonatais precoces e óbitos tardios.

Na Tabela 2 , está a descrição dos casos de rabdomioma que evoluíram para óbito. Dos três casos de óbito fetal, em dois casos ocorreu associação de massas tumorais gigantes, arritmia e hidropisia. Destes dois fetos com alteração de ritmo, um apresentou taquicardia supraventricular e o outro feto com 15 semanas apresentou bloqueio atrioventricular tipo 2:1. Apenas um caso apresentou massa tumoral gigante, sem arritmia ou hidropisia.

**Tabela 2:** Descrição dos casos de rabdomioma que evoluíram para óbito

Casos	IG-Dx	Tumor ≥ 20mm	Arritmia	Hidropisia	Tipo de óbito
1	35	N	S	N	ONNP
2	25	S	S	S	OF
3	39	S	N	N	ONNP
4	33	S	S	N	OT
5	33	N	N	S	ONNP
6	32	S	N	N	ONNP
7	13	N*	S	S	OF
8	34	N	N	N	OT
9	28	S	N	S	OF
10	34	S	N	S	OT
11	36	N	N	S	ONNP

N: não; S: sim; IG-DX: idade gestacional no diagnóstico de tumor; ONNP: óbito neonatal precoce; OF: óbito fetal; OT: óbito tardio; tumor gigante em feto com idade gestacional precoce.

Dos cinco óbitos neonatais precoces, dois deles foram submetidos à exérese do tumor e evoluíram com arritmia no pós-operatório. Os outros três pacientes apresentaram morte súbita antes da alta hospitalar.

Dos três óbitos tardios, em um paciente identificou-se como causa de óbito aos 13 dias de vida o acometimento hemodinâmico grave em rabdomioma gigante que impossibilitou a extração cirúrgica; o segundo óbito aos seis meses de vida foi por morte súbita em casa, com hipótese diagnostica de arritmia; e o terceiro óbito ocorreu aos 16 anos de idade devido às consequências debilitantes graves da esclerose tuberosa por acometimento neurológico, internações recorrentes e evolução para sepse.

A Figura Central apresenta a curva de Kaplan-Meier da probabilidade de sobrevida global (SG) acumulada dos pacientes com diagnóstico ecocardiográfico fetal de rabdomioma agrupados em três diferentes décadas. Observa-se uma melhora da sobrevida da década de 1990 para as demais décadas de 2000. A taxa de SG no primeiro período (1991 – 2000) foi de 54,5%, para o segundo período a taxa de SG foi de 91,7%, enquanto no último período a taxa de SG foi de 100% (p=0,002).

A associação das características clínicas e ecocardiográficas de fetos com os desfechos está demonstrada na tabela 3 . A hidropisia esteve presente em 14,3% dos casos e esteve associada ao óbito (p < 0,001).

**Tabela 3:** Associação entre as características clínicas e ecocardiográficas de fetos com diagnóstico de rabdomiomas e os desfechos

		Desfecho		p
		Vivo	Óbito	
**Histórico familiar**				1,00
	Não	43 (82,7)	9 (81,8)	
	Sim	9 (17,3)	2 (18,2)	
**Hidropisia**				<0,001
	Não	49 (94,2)	5 (45,5)	
	Sim	3 (5,8)	6 (54,5)	
**Arritmia**				0,36
	Não	34 (79,1)	2 (33,3)	
	Sim	9 (20,9)	4 (66,7)	
**Número de tumores**				0,67
	Único	13 (25,0)	1 (11,1)	
	Múltiplos	39 (75,0)	8 (88,9)	
**Tamanho de tumor**				0,51
	< 20mm	25 (58,1)	5 (45,5)	
	> 20mm	18 (41,9)	6 (54,5)	
**Esclerose tuberosa**				1,00
	Não	12 (27,9)	0	
	Sim	31 (72,1)	1 (100)	

* *Teste Exato de Fisher.*

## Discussão

Embora os tumores cardíacos sejam raros, o número de casos detectados vem aumentando significativamente durante as últimas décadas, não só devido à melhora do nível de rastreamento pelo ultrassom, mas também pelas melhorias técnicas dos equipamentos de imagem.^10 , 13 , 15 , 16^

O ecocardiograma fetal com diagnóstico de tumor cardíaco fetal foi realizado após uma triagem inicial e suspeita clínica de tumor. A incidência de tumores cardíacos fetais é de 0,009% em rastreamentos ultrassonográficos de baixo e alto risco e aumenta para 0,2% em centros de referencia em cardiologia pediátrica.^1^ Em nosso estudo, 8 em cada 10000 gestantes avaliadas tiveram diagnóstico fetal de rabdomioma.

Os principais achados do presente estudo envolvendo casuística de rabdomiomas cardíacos fetais foram: o rabdomioma é o tipo mais frequente (85%) de tumor cardíaco; apresenta alta morbidade (79,3%) e mortalidade geral relevante (17,4%), sendo a presença de hidropisia fetal preditiva de óbito.

Apesar de os rabdomiomas serem considerados histologicamente benignos, sua morbidade reside no fato de serem associados à esclerose tuberosa, doença profundamente debilitante, responsável por causar convulsões em 80% dos pacientes (incluindo espasmos infantis em aproximadamente 1/3) e atraso no desenvolvimento (incluindo transtorno do espectro autista) em mais de 60% dos casos.^14^ Embora em nossa série, a associação entre rabdomioma cardíaco e esclerose tuberosa tenha sido de 72,7%, semelhante aos resultados previamente descritos, ^8 - 14^ reconhecemos que este número poderia ser maior se tivéssemos tido acesso aos métodos de diagnóstico genético pela análise de DNA para os genes TSC1 e TSC2.^21 - 24^ Por limitações técnicas e econômicas, apenas quatro casos de nossa série foram confirmados geneticamente.

O uso de inibidores de mTOR nos casos de rabdomiomas com acometimento hemodinâmico já é uma realidade,^25 - 34^ com excelentes resultados não somente na redução das dimensões do tumor, como também no controle de sintomas neurológicos debilitantes da esclerose tuberosa. Em 2006 surgiu o primeiro relato de uso de inibidores de mTOR em pacientes com esclerose tuberosa.^31^ Em nossa população estudada, apenas três casos utilizaram o inibidor de mTOR, com regressão significativa das lesões após duas semanas de terapia.

A taxa de mortalidade geral foi de 17,4%. A presença de hidropisia fetal foi a única variável preditiva de óbito. Interessante foi a observação da melhora da taxa de sobrevida pós-natal nas últimas décadas, que subiu de 64,3% entre 1991 e 2000 para 91,7% entre 2001 e 2010 e para 87,5% entre 2011 e 2021. Parte desse aumento pode ser atribuído aos avanços no manejo e tratamento dos tumores cardíacos no período perinatal e a longo prazo.

Apesar de alguns estudos associarem a arritmia e o tamanho das massas tumorais como fatores de mau prognóstico,^4 , 8^ observamos que, isoladamente, esses fatores não contribuíram para a piora do prognóstico, mas sim quando evoluíram para um quadro de insuficiência cardíaca fetal grave, em sua maior expressão representada pela hidropisia fetal.

Em um dos casos de óbito, o diagnóstico de rabdomioma foi realizado com 13 semanas de gestação. Este feto apresentava bloqueio atrioventricular 2:1 e histórico familiar de um parente de primeiro grau portador de esclerose tuberosa oligossintomática (irmã). Este histórico confirmou a presença de uma doença genética familiar grave que embora com expressividade variável, apresentou alto grau de penetrância, impactando no planejamento de futuras gestações.

As limitações do nosso estudo incluem o número relativamente pequeno de casos para a análise mais elaborada em alguns aspectos, como a associação das características ecocardiográficas do tumor com os resultados e a análise de sobrevida. Alem disso, o teste genético em pacientes com esclerose tuberosa e a confirmação anatomopatológica dos tumores foram realizados em um número pequeno de pacientes. Entretanto, esses fatores não limitaram as conclusões obtidas.

## Conclusão

A presença de hidropisia fetal teve impacto na mortalidade, sendo fator importante para aconselhamento e estabelecimento de prognóstico. O número relevante de pacientes com seguimento tardio, assim como a ausência de interrupção nesta série quando se compara com a maioria das publicações que apresentam esse viés, faz com que este estudo se aproxime da história natural da doença, fato não descrito anteriormente na literatura médica.
