# Supplementary material for: Resultados Perinatais e Seguimento em Longo Prazo de Tumores Cardíacos Fetais: Estudo de Coorte Histórica de 30 Anos
Source: Arq Bras Cardiol. 2024 Mar 13;121(1):e20220469. [Article in Portuguese] doi: 10.36660/abc.20220469 (PMC11081142; doi:10.36660/abc.20220469)
Supplement: Translation

# Translation: Perinatal Results and Long-Term Follow-Up of Fetal Cardiac Tumors: A 30-Year Historical Cohort Study

**Authors:** Fabricio Marcondes Camargo, Maria de Lourdes Brizot, Rossana Pulcineli Vieira Francisco, Werther Brunow de Carvalho, Nana Miura Ikari, Stella Verzinhasse Peres, Marco Antônio Borges Lopes, Lilian Maria Lopes

**Affiliations:** 1 São Paulo SP Brazil Hospital das Clínicas da Faculdade de Medicina da Universidade de São Paulo, São Paulo, SP – Brazil; 2 São Paulo SP Brazil Instituto do Coração do Hospital das Clínicas da Faculdade de Medicina da Universidade de São Paulo, São Paulo, SP – Brazil; 3 São Paulo SP Brazil Universidade de São Paulo – Faculdade de medicina, São Paulo, SP – Brazil; 4 São Paulo SP Brazil ECOKID – Cardiologia e Ecocardiografia Fetal Pediátrica e Materna, São Paulo, SP – Brazil

**Keywords:** Heart Neoplasms, Echocardiography, Prenatal Diagnosis, Rhabdomyoma

## Abstract

**Background:**

This was a 30-year retrospective cohort study that approximates closely to the natural history of cardiac tumors diagnosed in the fetus, since there was no case of pregnancy interruption

**Objective:**

To assess morbidity and mortality in the perinatal period and at long term in fetuses diagnosed with cardiac tumor. Our secondary objective was to assess the evaluating factors of perinatal and postnatal results.

**Methods:**

This was a retrospective cohort study with 74 pregnant women with an echocardiographic diagnosis of fetal cardiac tumor at two referral centers between May 1991 and November 2021. A descriptive analysis was performed, and data were expressed as absolute (n) and relative (%) frequencies, median and interquartile range. Fisher's exact test was used to evaluate the association of echocardiographic characteristics and clinical manifestations with perinatal and postnatal results. Global survival was calculated using the Kaplan-Meier method and the curves were compared by the log-rank test. The time of follow-up, calculated in months, corresponded to the time elapsed from hospital discharge to current status (survived/ censoring or death). The level of significance was set at 5% (p<0.05).

**Results:**

Rhabdomyoma is the most common type of cardiac tumor (85%), with a high morbidity (79.3%) and overall mortality of 17.4%. The presence of fetal hydrops was a predictor of death.

**Conclusion:**

The presence of fetal hydrops had an impact on mortality, and hence is an important factor in counselling and determining the prognosis. Most deaths occurred before hospital discharge.

## Introduction

Primary cardiac tumors are rarely found in utero or in the postnatal period. The incidence of these tumors is 0.009% in low and high-risk ultrasound and increases to 0.2% in referral centers for pediatric cardiology.^1^

More than 90% of primary cardiac tumors are benign and originate in the pericardium or in the myocardium.^2^Of the five histological types of congenital cardiac tumors, rhabdomyomas account for 60%-86% of tumors, followed by teratomas and fibromas, whereas hemangiomas and hamartomas are extremely rare.^3^

The presence of cardiac tumors in the fetus may cause important hemodynamic changes that affect both fetal and postnatal morbidity and mortality, including hydrops, arrythmia and ventricular inflow and outflow obstruction.^4 - 7^Besides, the prevalence of tuberous sclerosis associated with fetal cardiac rhabdomyomas is high, estimated to be between 50 and 80%.^4 , 8 - 13^Therefore, the detection of cardiac tumor is an important marker in the investigation of tuberous sclerosis, which is a genetic disease with neurological, renal, ophthalmological and dermatological manifestations in addition to cardiac ones.^14 , 15^Significant morbidity, especially when associated with tuberous sclerosis, is related to changes in the central nervous system, including convulsions, developmental delay, and autism spectrum disorder.^14^

In the last decade, fetal diagnosis of primary cardiac tumors has tremendously increased. This is partially due to advances in imaging techniques that enable accurate diagnosis and histological classification of different tumors.^10 , 13 , 15 , 16^

Few studies have evaluated long-term and perinatal outcomes of fetuses with cardiac tumors.^4 , 8 - 12^The present study aims to help to fill this gap, by presenting data of morbidity and mortality, and influencing factors of these outcomes. Besides, the major contribution of this series is to describe a scenario that approximates closely to the natural course of disease, since there was no case of pregnancy interruption.

## Methods

This was a retrospective cohort study conducted between May 1991 and November 2021, involving fetuses with an echocardiographic diagnosis of cardiac tumors at two referral centers for fetal echocardiography (Division of Echocardiography and Fetal Cardiology of the Department of Obstetrics of the General Hospital of the University of Sao Paulo Medical School, HCFMUSP and the Ecokid education and research institute). This study was approved by the ethics committee of the hospital (CAAE: 93022218.9.0000.0068).

During the 30-year study period, 30,915 pregnant women were evaluated at the Division of Echocardiography of the HCFMUSP, and 46,671 at Ecokid, with 74 fetuses with cardiac tumors.

Inclusion criteria included fetuses with confirmed postnatal echocardiographic (in live births) or anatomopathological diagnosis (fetal deaths). At the two institutions, echocardiograms were performed by the same examiners who worked at both centers following the same follow-up protocol. All echocardiograms were performed and revised by two pediatric cardiologists, experienced in fetal echocardiography (F.M.C and L.M.L.).

Tumors were classified according to echocardiographic features in terms of echogenicity (homogenous or cystic), tumor borders (well-defined or irregular), number (solitary or multiple), location and size (larger or smaller than 20mm). Rhabdomyomas were defined as homogeneous masses of variable size, multiple (mostly) or solitary, with no flow within them. Fibroma was defined as hyperechogenic solitary masses, with calcification and cystic degeneration. Teratomas showed complex echogenicity with cystic and solid components associated with calcifications, and the only case of hemangioma showed, as described in the literature, to be vascularized and localized in the right atrium. Figure 1 shows examples of the types of fetal cardiac tumors diagnosed by fetal echocardiography.

**Figure 1:** Examples of the types of fetal cardiac tumors diagnosed by fetal echocardiography. RV: right ventricle; LV: left ventricle; RA: right atrium; LA: left atrium; AO: aorta.

In addition, hemodynamic repercussion of the tumor was assessed, particularly for the presence of hydrops, ventricular inflow and outflow obstruction, and arrythmia. Fetal hydrops was defined as the presence of abnormal fetal fluid collections in two or more cavities in the absence of alloimmunization.^17^To quantify the progression of fetal heart failure, we used the score proposed by James Huhta,^18^which included hydrops, cardiothoracic ratio, cardiac function and arterial and venous waveforms.

The ultrasound devices used in the study were Toshiba 270, HDI 3000 (Advanced Technology Laboratories, Bothell, WA, USA) and GE ultrasound systems (Voluson 730 Expert, GE Voluson E8, S6 expert and E10). Both sector and convex (5.0 and 3.5MHz) probes were used. The fetal heart was examined by two-dimensional, M-mode, (pulsed-wave) spectral Doppler, and Doppler color flow imaging. Cardiac rhythm was determined by analysis of mechanical systole of the atrial and ventricular walls by pulsed Doppler.

After the diagnosis of fetal tumor, pregnant women without hemodynamic compromise were followed-up monthly, and those who had hemodynamic compromise, such as hydrops and arrythmia, were followed-up weekly or fortnightly, according to case severity. They also received high-risk prenatal care. Delivery was planned to occur at 39 weeks’ gestation and the type of delivery depended on obstetric indication.

Diagnostic confirmation of the tumors was carried out in the neonatal period by echocardiography and magnetic resonance imaging, in addition to anatomopathological analysis of patients who underwent surgery or died. Tumors were classified following the 2015 WHO classification of tumors of the heart and pericardium.^18 , 19^

Patients with a confirmed diagnosis in the postnatal period received outpatient care with a pediatric cardiologist and patients who had a diagnosis of tuberous sclerosis were also followed by pediatric neurologists.

Morbidity was defined as need for intensive care unit (ICU) admission, postnatal treatment with antiarrhythmic drugs, anticonvulsants or mTOR inhibitors (everolimus and sirolimus), need for postnatal surgery, neurological impairment, and diagnosis of tuberous sclerosis. As neurological impairment, the presence of the following conditions was considered: tuberous sclerosis, convulsion, neurodevelopmental disorders. Data on the diagnosis of tuberous sclerosis was obtained patients’ medical records and the follow-up form used in the interview.

### Statistical analysis

A descriptive analysis of the data was performed using absolute (n) and relative (%) frequencies. The denominator indicates the number of cases related to the variable of interest.

In the inferential analysis of data, only fetuses with rhabdomyoma were included. Distribution of quantitative variables was analyzed by the Komolgorov-Smirnov test for normality. As data did not show a normal distribution, data were described as median and interquartile range. The binomial test was used to identify differences in sex distribution. Independent variables related to death and tuberous sclerosis were compared using the Fisher's exact test. Global survival (GS) was calculated by the Kaplan Meier and the curves were compared using the log-rank test. The time of follow-up, calculated in months, corresponded to the time elapsed from hospital discharge to current status (survived/ censoring or death).

The significance level was set at 5% (p<0.05). Data were analyzed using the SPSS software for Windows, version 20.

## Results

Indications for echocardiogram at the time fetal rhabdomyoma was diagnosed were suspicion of tumor (n=42, 68.8%), fetal arrhythmia (n=9, 14.7%), suspicion of congenial heart disease (n=7, 11.5%), routine examination (n=3, 5%).

Of the 74 cases described, 12 (16.2%) underwent biopsy, which confirmed the histological diagnosis, seven after death and two after surgery. However, the absence of a histological study does not invalidate the diagnosis, since studies have shown that diagnosis is mostly made by imaging, clinical and genetic tests.^20^Besides, the use of an invasive diagnosis procedure is not risk-free.

Eleven cases of rare tumors were found: six (8.1%) fibromas, four (6.4%) teratomas and one (1.3%) hemangioma. Rhabdomyomas were the most common type, with a total of 63 fetuses (85.1%).

### Fibroma

There were six confirmed cases of fibroma, two of them progressed to death. In both cases, the tumor was large and located on the posterior wall of the left ventricle. The first case died at the age of one year while waiting for a heart transplant, and the second died three days after birth ( Figure 1a ), before hospital discharge. The other four cases survived, did not undergo surgery and are still in clinical follow-up.

### Teratoma

Of the four cases of teratoma, three died (two fetuses and one neonatal case). All cases showed important pericardial effusion and fetal hydrops; one underwent puncture that led to expansion of tumor mass and death two days following the procedure, at 25 weeks of gestation. One patient is alive and asymptomatic after tumor excision ( Figure 1b ).

### Hemangioma

The only case of hemangioma, diagnosed at 24 weeks of gestation, was born at 38 weeks’ gestation in a referral hospital for pediatric cardiac surgery. It was a vascular tumor, located in the right atrium ( Figure 1c ), fed by a fistula in the right coronary artery ( Figure 2 ). The patient is well, followed by a pediatric cardiologist, without medications.

**Figure 2:** Right atrial hemangioma fed by a fistula in the right coronary artery. RA: right atrium; LA: left atrium.

### Rhabdomyoma

Rhabdomyomas were the most common type of tumor, diagnosed in 63 fetuses (85.1%). Median gestational age at the diagnosis of rhabdomyomas was 32 (13-39) weeks. Median mother age at the diagnosis was 29 (15-39) years. Family history of tuberous sclerosis was present in 18% (11/63) of patients diagnosed with rhabdomyoma.

Most tumors (92.3%) were located in the ventricles, most were multiple (77%) and approximately 45% had a diameter greater than 20mm ( Figure 1.d ). Fifty-eight were born alive, with a median gestational age of 38 (37-39) weeks, and the most common type of delivery was cesarean (81.8%; 45/55). The frequency of male and female newborns was not different, 52.5% (31/59) and 47.5% (28/59) respectively (p = 0.79). An Apgar score less than or equal to seven was observed in 7.7% (4/52) of live-born infants.

Fifty patients (83.3%) experienced some type of morbidity. Median hospital stay was 10 (5-19) days, and most patients were admitted to neonatal ICU. Of these patients (n=50), 16.1% required surgery and 88.7% were discharged.

Regarding the use of medications, 51.9% (27/52) required treatment after birth. Four needed antiarrhythmic drugs, 20 required drugs with an action on the central nervous system (e.g., anticonvulsants and antipsychotics) and three received mTOR inhibitors such as everolimus and sirolimus ( Figure 3 ). Neurological impairment was recorded in the medical chart of 77.8% (35/45) of patients, as neurodevelopmental disorders and convulsions, often concomitant. Information regarding the follow-up period was obtained from 53 cases with a median follow-up time of 70 (12-174,3) months. Table 1 shows the distribution of clinical and ultrasonographic characteristics of patients with fetal echocardiographic diagnosis of rhabdomyoma.

**Figure 3:** Patient undergoing treatment with mTOR inhibitor before and after birth. RV: right ventricle; LV: left ventricle; RA: right atrium; LA: left atrium.

**Table 1:** Distribution of clinical and ultrasonographic characteristics of patients with fetal echocardiographic diagnosis of rhabdomyoma

Characteristics		N, %
**Sex**		
	Female	28/59 (47.5)
	Male	31/59 (52.5)
**Family history**		11/63 (17.5)
	Hydrops	9/63 (14.3)
	Arrythmia	13/49 (26.5)
**Number of tumors**		
	Solitary	14/61 (23.0)
	Multiple	47/61 (77.0)
**Tuberous sclerosis**		32/44 (72.7)
**Localization**		
	Atrial	4/63 (6.3)
	Ventricular	58/63 (92.1)
	Atrial and ventricular	1/63 (1.6)
**Diameter > 20mm**		24/54 (44.4)
**Outcome**		
	Survived	52/63 (82.5)
	Died *	11/63 (17.5)

The denominator indicates the number of cases related to the variable of interest;

* fetal deaths, early neonatal deaths and late deaths.

Table 2 describes the progression of patients with rhabdomyoma who progressed to death. In two of the three cases of fetal death, an association of giant tumor, arrythmia and hydrops was observed. Of these fetuses that presented altered cardiac rhythm, one had supraventricular tachycardia and the other fetus, at 15 weeks of age, presented 2:1 atrioventricular block. Only one patient had giant tumor, with no arrythmia or hydrops.

**Table 2:** Description of the cases of rhabdomyoma that progressed to death

Cases	Gestational age at diagnosis	Tumor ≥ 20mm	Arrythmia	Hydrops	Type of death
1	35	N	Y	N	END
2	25	Y	Y	S	FD
3	39	Y	N	N	END
4	33	Y	Y	N	LD
5	33	N	N	S	END
6	32	Y	N	N	END
7	13	N *	Y	S	FD
8	34	N	N	N	OT
9	28	Y	N	S	FD
10	34	Y	N	S	LD
11	36	N	N	S	END

N: no; Y: yes; END: early neonatal death; FD: fetal death; LD: late death;

* giant tumor at early gestational age.

Two of the five early neonatal deaths underwent tumor excision and developed arrhythmia after surgery. The other three patients had sudden death before being discharged.

Three cases of late deaths were identified; one patient died at 13 days of age due to severe hemodynamic impairment and giant rhabdomyoma which made surgical excision difficult; the second patient died at six months of age due to sudden death, with diagnostic hypothesis of arrhythmia; the third patient died at the age of 16 years due to severe debilitating consequences of tuberous sclerosis, recurrent hospitalizations and development of sepsis.

Figure 4 (Central Illustration) presents Kaplan Meier curves of accumulated global survival (GS) probability of patients with echocardiographic diagnosis of rhabdomyoma grouped into three decades. There was an improvement in survival from 1990 to decades of 2000. In the first period (1991-2000), GS rate was 54.5%, in the second period 91.7%, and in the third period 100% (p=0.002).

Association of clinical and echocardiographic characteristics of the fetuses with the outcomes is described in Table 3 . Hydrops was present in 14.3% of the cases and associated with death (p=0.001).

**Table 3:** Association of clinical and echocardiographic characteristics of the fetuses diagnosed with rhabdomyomas and outcomes

		Outcome		p
		Survival	Death	
**Family history**				1.00
	No	43 (82.7)	9 (81.8)	
	Yes	9 (17.3)	2 (18.2)	
**Hydrops**				<0.001
	No	49 (94.2)	5 (45.5)	
	Yes	3 (5.8)	6 (54.5)	
**Arrhythmia**				0.36
	No	34 (79.1)	2 (33.3)	
	Yes	9 (20.9)	4 (66.7)	
**Number of tumors**				0.67
	Solitary	13 (25.0)	1 (11.1)	
	Multiple	39 (75.0)	8 (88.9)	
**Tumor size**				0.51
	< 20mm	25 (58.1)	5 (45.5)	
	> 20mm	18 (41.9)	6 (54.5)	
**Tuberous sclerosis**				1.00
	No	12 (27.9)	0	
	Yes	31 (72.1)	1 (100)	

* Fisher's exact test.

## Discussion

Fetal echocardiogram with diagnosis of fetal heart tumor was performed after initial screening and clinical suspicion of tumor. The incidence of fetal heart tumors is 0.009% in low- and high-risk ultrasound screenings and increases to 0.2% in pediatric cardiology reference centers.^1^In our study, 8 out of every 10,000 pregnant women evaluated had a fetal diagnosis of rhabdomyoma.

Although cardiac tumors are rare, the number of diagnosed cases has significantly increased over the last decades, not only due to improvements in the performance of ultrasound screening, but also to technical advances in imaging devices.^10 , 13 , 15 , 16^

The main findings of the present study, involving cases of fetal cardiac rhabdomyomas were: rhabdomyoma is the most common (85%) type of cardiac tumor, with a high morbidity (79.3%) and relevant overall mortality (17.4%), with the presence of fetal hydrops as a predictor of death.

Although rhabdomyomas are histologically benign, their morbidity is due to the association with tuberous sclerosis, a severely debilitating condition, responsible for causing convulsion in 80% of patients (including autism spectrum disorder) in more than 60% of cases.^14^Although we found an association between cardiac rhabdomyoma and tuberous sclerosis of 72.7% in our series, which is similar to previously reported results,^8 - 14^we understand that this number could be higher, if we could have performed DNA analysis for the TSC1 and TSC2 genes in the diagnosis.^21 - 24^Due to technical and economical limitations, only four cases in our series were genetically confirmed.

The use of mTOR inhibitors in rhabdomyoma with hemodynamic compromise is already a reality,^25 - 34^with excellent results not only in reducing tumor size, but also in the control of debilitating neurological symptoms of tuberous sclerosis. In 2006, the first report of the use of mTOR inhibitors in patients with tuberous sclerosis appeared.^31^In our studied population, mTOR inhibitors were used in only three cases, with significant regression of the lesions after two weeks of therapy.

The overall mortality rate was 17.4%. The presence of hydrops fetalis was the only variable predictive of death. Interestingly, postnatal survival rates have increased in the last decades, from 64.3% between 1991 and 2000 to 91.7% between 2001 and 2010, and to 87.5% between 2011 and 2021. Part of such increase may be attributed to advances in the management and treatment of cardiac tumors in the perinatal period and in long term.

Although some studies have correlated arrhythmia and the size of tumor masses with poor prognosis,^4 , 8^we found that these factors alone did not contribute to a worse prognosis, but rather, it happened when they progressed to severe fetal heart failure, mostly manifested by fetal hydrops.

In one of the cases of death, the diagnosis of rhabdomyoma was made at 13 weeks of gestation. This fetus had 2:1 atrioventricular block and a family history of a first-degree relative with oligosymptomatic tuberous sclerosis (sister). This confirmed the presence of a severe familial genetic disease, which despite variable expressivity, has a high level of penetrance, affecting the planning of pregnancy.

Limitations of our study include the relatively small number of cases that limited the possibility of some more detailed analyses, such as the association of echocardiographic characteristics of the tumor with the results, and a survival analysis. Besides, genetic tests in patients with tuberous sclerosis and anatomopathological confirmation of the tumors were carried out in a small number of patients; however, these factors did not affect our conclusions.

## Conclusion

The presence of fetal hydrops had an impact on mortality, and hence is an important factor in counselling and determining the prognosis. With the relevant number of patients in long-term follow-up and the lack of interruption of the series as compared with most of publications with this bias, the present study approximates closely to the natural history of the disease, which had not been previously described in the medical literature.
